# Current Status and Future Trends in Myocarditis Related to the COVID-19 Vaccines: A Visual and Bibliometric Analysis

**DOI:** 10.2174/0115701611287623250107074054

**Published:** 2025-01-17

**Authors:** Youao Zhang, Mengjia Wang, Jieyan Wang

**Affiliations:** 1 The First School of Clinical Medicine, Southern Medical University, Guangzhou, China;; 2 Department of Urology, People's Hospital of Longhua, Shenzhen, Guangdong, 518109, China

**Keywords:** COVID-19 vaccines, myocarditis, hotspots, frontiers, bibliometrics

## Abstract

**Aims:**

This study aims to conduct a bibliometric and visual analysis of published studies on myocarditis and coronavirus disease 2019 (COVID-19) vaccines.

**Background:**

The widespread epidemic of COVID-19 has caused millions of deaths and profoundly affected the global medical landscape. Studies on COVID-19 vaccination and related myocarditis have also increased significantly.

**Objective:**

To analyze the current status and trends of myocarditis and COVID-19 vaccine research by bibliometric and to elucidate research hotspots and frontiers.

**Methods:**

Based on the Web of Science Core Collection SCI-Expanded database, we utilize Excel 2019 and visualization analysis tools VOSviewer, Co-Occurrence13.2 (COOC13.2), Citespace, HistCite, and Scimago Graphica for analysis.

**Results:**

Our study encompassed a total of 389 relevant articles, and we observed a consistent upward trend in the number of publications over time, indicating the growing interest in this subject. Among the countries and regions contributing to this body of literature, the United States emerged as the leading publisher, with Harvard Medical School being the most prominent institution associated with these studies. Notably, Matthew E. Oster from the United States emerged as one of the prominent authors in this field. Hotspot research and frontier areas include myocarditis and the different types of COVID-19 vaccines (*e.g*., mRNA vaccines, adenovirus vector vaccines, inactivated vaccines), the development of new vaccines in reducing the incidence and sequelae of COVID-19 without an increased incidence of myocarditis, and relief of vaccine hesitancy.

**Conclusion:**

Research on myocarditis and the COVID-19 vaccines has grown rapidly. Our research results can help researchers grasp the current status of myocarditis related to the COVID-19 vaccine research and find new research directions in the future.

## 1 INTRODUCTION

COVID-19 has led to a global pandemic and overwhelmed global healthcare systems [1]. A safe and effective vaccine has emerged as the most cost-effective way to prevent and slow the spread of COVID-19 infection compared to drugs and other treatments [2]. Vaccines can play an important role in improving population immunity, preventing serious diseases, and reducing ongoing health crises [3]. Vaccination has been declared as one of the best approaches against novel coronaviruses [4]. We checked on the WHO website (https://covid19.who.int/) that as of 25 April 2023, 5,104,300,510 people had been fully vaccinated worldwide. However, due to widespread vaccination and subsequent vaccine controversy, safety monitoring of the COVID-19 vaccine is critical to ensure safety, maintain trust, and inform policy [5]. The estimated incidence of post-vaccination myocarditis is 2.13 cases per 100,000 population, and most cases of myocarditis are mild or moderate in severity [6, 7]. However, other studies have shown that the benefit-risk assessment of COVID-19 vaccination showed that cases of myocarditis are rare, symptoms are mild and resolve rapidly, and are well-balanced in all age and gender groups [6-9]. In the face of different views on the possibility of myocarditis caused by COVID-19 vaccination, we hope to understand the relationship between different generations of COVID-19 vaccines and myocarditis more intuitively and comprehensively through bibliometric methods to provide reference for solving vaccine hesitancy, prevention of sequelae and vaccine improvement.

The bibliometric analysis employs mathematical and statistical techniques to qualitatively or quantitatively examine the distribution, structure, quantity, and evolution of bibliographic information. This method holds significant value in characterizing the existing landscape across different research disciplines, tracking publishing trends, and assessing the scientific accomplishments of researchers, institutions, and countries. Furthermore, it enables the identification of future research hotspots, academic frontiers, and knowledge maps, thereby providing researchers and clinicians with a comprehensive understanding of the current state of development within a specific research domain. By leveraging bibliometric analysis, we can gain insightful insights into the intricate dynamics that shape the research ecosystem and drive advancements in the field. Moreover, bibliometrics have been widely used in COVID-19, vaccines and cardiovascular disease [2-4, 10-13], however, no bibliometrics of COVID-19 vaccinations related myocarditis were published. VOSviewer is also a commonly used software in various fields of bibliometrics [3, 10-17], while COOC is a software developed by Chinese scholars for bibliometrics and scientific mapping and is constantly iterated [10]. COOC software has also been increasingly used in SCI-E articles [10, 14, 15, 18]. Additionally, Citespace, HistCite, and Scimago Graphica are also often used in bibliometrics [3, 11, 12, 19-21]. By conducting a bibliometric analysis of the existing literature, we aim to provide valuable insights into the current landscape of myocarditis research in the context of COVID-19 vaccines. We hope that our findings will contribute to a better understanding of this important topic and aid future research efforts in this area.

## 2 METHODS AND MATERIALS

### 2.1 Data Retrieval Strategy, Data Extraction and Cleaning

The objective of this paper is to investigate the correlation between myocarditis and the COVID-19 vaccine. To gather relevant data, we utilized the Web of Science Core Collection, a highly regarded and comprehensive database that encompasses more than 12,000 high-quality journals. Thus, we have chosen the Web of Science Core Collection SCI-Expanded (SCI-E) database as the data source for our research, and selected the advanced search, the search formula: TS= (2019-Coronavirus or 2019-CoV or Coronavirus 2019 or ncovid19 or Novel Coronavirus 2019 or SARS coronavirus two or SARS-CoV-2 or Severe Acute Respiratory Syndrome Coronavirus 2 or COVID-19 or Coronavirus Disease 2019 or COVID* or 2019-nCoV or nCoV-19) AND TS= myocarditis AND TS = (vacc* or immuniz*). The retrieval date was from January 1, 2020, to December 31, 2022. The screening process is detailed in Fig. (**1**).

### 2.2 Scientometric Analysis Methods

The 389 pieces of literature were exported in plain text format. We utilized Excel 2019 and visualization analysis tools VOSviewer, COOC13.2, Citespace, HistCite, and Scimago Graphica for overall trend analysis, synonym merging, frequency of countries/regions, institutions, and authors, cluster analysis of co-occurrence matrix, two-mode matrix, burst keywords map to explore the research hotspot and frontier direction of myocarditis and the COVID-19 vaccines.

## 3 RESULTS

### 3.1 Annual Analysis of Publication

Since the outbreak of COVID-19 at the end of 2019, the research on the COVID-19 vaccine and myocarditis has increased rapidly every year, with 7 in 2020 66 in 2021, and 316 articles in 2022, showing that the research in this field has increased rapidly.

### 3.2 Country/region, Institution, Author, and Journal Frequency Analysis

Through the frequency analysis of countries/regions (Table **1**), it can be seen that the USA is the country with the most research on the COVID-19 vaccine and myocarditis. Europe, North America, and Asia all have a large number of publications. The largest research institution is Harvard Medical School. Research institutions are mainly concentrated in the USA, Israel, and the UK. The most published author is Matthew E Oster from Emory University. The top three journals were Vaccines, Frontiers in Cardiovascular Medicine, and Vaccine. Furthermore, four of the top 10 journals are comprehensive journals (including two prestigious journals, the New England Journal of Medicine and the BMJ), two are vaccine journals, two are cardiovascular journals, a journal of immunology, and a journal of infectious diseases. The diverse categories of journals noticed the direction of COVID-19 vaccines and myocarditis. To a certain extent, the research on the COVID-19 vaccine and myocarditis involves many studies and has been paid attention to in many fields.

### 3.3 Institutions Authors, and Countries/Regions, Analysis of Cooperation

It can be seen from Fig. (**2A**) that the cooperation between China and England is the most extensive. Meanwhile, the USA also has close cooperation with Germany. In terms of institutions, Fig. (**2B**), shows the Tel Aviv University cooperates with Ben Gurion University of the Negev and the Hebrew University of Jerusalem five times, respectively. Three of them are from Israel. UCL and the University of Hong Kong have also collaborated five times. According to Fig. (**2C**), professors Ian Chi Kei Wong and, Francisco Tsz Tsun Lai, Patrick Ip each collaborated four times. Ip, Patrick and Gilbert T Chua, and Bruce Fireman, and Joshua T B Williams also have collaborated four times each, making them the authors with the most collaborations. In general, the cooperation among authors is relatively concentrated.

### 3.4 Citation Analysis

#### 3.4.1 Analysis of Citation

According to Table **2**, due to the sudden outbreak of COVID-19, the possible complications of COVID-19 vaccines are still in the exploratory stage. The most cited is a 2020 review by Elissa Driggin *et al*. published in the Journal of the American College of Cardiology (JACC), which focuses on describing the link that exists between patients with COVID-19 and those with pre-existing cardiovascular disease and how healthcare workers can protect themselves in the event of harm [22]. The second most cited is a review by Mohammad Madjid *et al*. published in JAMA Cardiology in 2020. The review mentions the search for specific vaccines and antiviral drugs against SARS-CoV-2 as a current research direction [23]. The third most cited article published article in Circulation by Peter P Liu *et al*. in 2020, focuses on the fact that COVID-19 usually involves the cardiovascular system early. Antibody testing and effective vaccines are needed to make COVID-19 history in the future in order to protect more people [24].

#### 3.4.2 Usage Rate Analysis in 180 Days

The frequency of usage within a 180-day period indicates the number of times the article has fulfilled a user's information requirements, as evidenced by their engagement with the full-length article through clicking links on the publisher’s website or saving the metadata for future reference. Although a high usage count cannot be immediately translated into a high number of citations, it has the advantage of novelty [25]. The number of 180-day use can reflect the current research hotspots and frontiers to a certain extent. According to Table **3**, the highest use of 180 days was in a guideline published by Ty J Gluckman *et al*. in JACC in 2022 on cardiovascular sequelae of COVID-19 such as myocarditis and other myocardial involvement, as well as acute sequelae of SARS-CoV-2 infection and considerations for athletes returning to competition [26]. Tied for first place is a review by Thibault Fiolet *et al*. in Clinical Microbiology and Infection in 2022. The main story is that all vaccines appear to be safe and effective tools against all variants of interest to prevent severe COVID-19, hospitalization, and death. Still, the quality of evidence varies depending on the vaccine considered, and despite rare serious adverse effects, the benefits of COVID-19 vaccination outweigh the risks [27]. In the third place is a review by Biykem Bozkurt *et al*. published in circulation in 2019. In this article, myocarditis is considered to be a rare complication of COVID-19 mRNA vaccination, particularly in young adults and adolescent males. Although rare cases of myocarditis have occurred, the benefit-risk assessment of COVID-19 vaccination shows a good balance across all age and sex groups. Therefore, COVID-19 vaccination is recommended for everyone aged ≥12 years [8].

#### 3.4.3 Citation Map Analysis

**Fig. (3):**
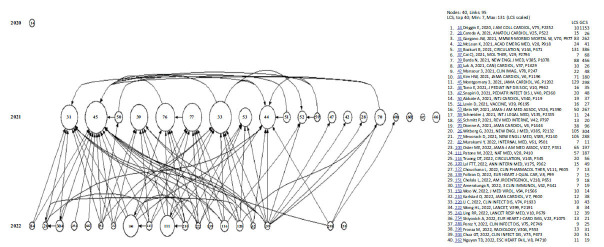
Citation map analysis.

We also performed co-citation analysis using HistCite. We mapped the citations of the top 40 articles in the LCS through HistCite (Fig. **3**). This chart shows how these articles are related to each other and how these articles refer to other articles. We can observe the centers of most of the connections in the included articles through HistCite's citation map. It can be divided into the citation center and the application center. Fig. (**3**) shows that the cited center is No. 45 in the figure (Montgomery J, 2021, JAMA Cardiol, V6, P1202) [28]. The cited literature center is No. 159 (Woo W, 2022, J Med Virol, V94, P1566) [29], which cites a large number of LCS 40 articles, and this article may be a good choice for a comprehensive understanding of this field.

### 3.5 Keywords Analysis

#### 3.5.1 Keywords Frequency and Co-occurrence Analysis

COOC13.2 was utilized to extract keywords, which were then combined synonymously. Finally, the top 50 keyword frequencies are presented in Fig. (**4A**). Keyword frequency serves as a crucial indicator that directly reflects the research content, research hotspots, and emerging direction of a field. In Fig. (**4B**), the presence of correlations among the keywords mentioned in the paper is depicted through their co-occurrence frequency. It is generally acknowledged that the more frequently two lexical pairs appear together in the same literature, the closer the relationship between these two topics can be inferred. According to Fig. (**4B**), excluding the center words of the articles such as COVID-19, SARS-CoV-2, vaccine, *etc*., we find that pericarditis, and myopericarditis are also more closely related to the center words.

#### 3.5.2 Keywords, Authors Analysis

To establish the relationship between authors, we utilized the coupling strengths of keywords within their works. Bimodal matrices were constructed based on the number of identical keywords shared between any two authors. By visualizing the correlation between authors and keywords, we can effectively uncover the structure of disciplinary knowledge centered around the main authors. This visualization chart, depicted in Fig. (**5A**), offers a clear and direct understanding of the researchers' research content and facilitates the identification of potential collaborations between authors within the same research field. Figs. (**5A**, and **B**) provide an intuitive display of the authors' research focus. For instance, Professor Matthew E. Oster's extensive research spans various topics such as COVID-19, SARS-CoV-2, myocarditis, pericarditis, vaccines, adolescents, and vaccination. Additionally, Figs. (**5A** and **5B**) allow us to identify common research fields among authors. For instance, Professor Matthew E. Oster's extensive research spans various topics such as COVID-19, SARS-CoV-2, myocarditis, pericarditis, vaccines, adolescents, and vaccination. Additionally, Figs. (**5A**, **B**) allow us to identify common research fields among authors, for example, Arthur Shiyovich, Guy Witberg, Ashraf Hamdan, Yaron Aviv, and Ran Kornowski. These five authors have similar research fields.

#### 3.5.3 Keywords Cluster Analysis

Cluster analysis can give us a better understanding of the research direction of the topic and a more systematic and comprehensive understanding of the topic. Citespace can be well used for cluster analysis, strategic coordinate graph analysis, and keyword co-occurrence structure analysis, so we used the keywords and title words in Citespace software for cluster analysis and got Fig. (**6**). Fig. (**6A**) has 10 clusters. Fig. (**6B**) has cluster # 1 ending story #2 following COVID-19 mRNA vaccination #3 health system #4 recent advance #5 CoV-2 infection#6 COVID-19 mRNA vaccine #7autoinflammatory condition #8 cardiovascular system # 9 characteristics efficacy. Fig. (**6C**) has cluster #0 cardiovascular diseases # 1 influencing factor # 2 MIS # 3 COVID-19 mRNA vaccine #4 cardiovascular system #5 coronavirus disease #6 mRNA vaccine #7 COVID-19 vaccine-associated myocarditis.

#### 3.5.4 Keywords, Time Analysis

Keywords and time analysis can provide valuable insights into their evolution and offer a better understanding of the research frontiers within a given topic. In this study, COOC13.2 software was employed to draw Fig. (**7**), which effectively illustrates the changing trends of research topics in the field over time. Fig. (**7A**) specifically focuses on the annual keyword mutation, which can better grasp a comprehensive examination of the predominant research themes in each year. This analysis provides valuable references for future research and development of the industry as it identifies recent mutation words that represent emerging hot issues. By setting the interception frequency to 20 through COOC13.2, Fig. (**7A**) was obtained, highlighting the most significant annual keyword mutations. In Fig. (**7B**), each circle represents a keyword, with the larger the circle the higher the frequency of t that keyword in the year it first appeared in the analyzed dataset. Once the keyword appears, it is fixed to the year of first appearance; although it may continue to appear in subsequent papers, it is only depicted in the figure for its first appearance year. If the keyword reoccurs in later years, its frequency is added to the position where the keyword first appeared, and the frequency is incremented accordingly. The COOC13.2 software was utilized to plot Fig. (**7B**), which visualizes the evolution of research themes over time to reflect the trend of the development in the domain. Overall, the combination of Fig. (**7A** and **7B**) offers a comprehensive understanding of how research topics have evolved, providing insights into both the annual shifts and the long-term trend of research theme development within the field.

## 4 DISCUSSION

### 4.1 General Information

The number of studies on myocarditis related to the COVID-19 vaccine has shown a sharp upward trend. It may be related to the increased availability and usage of the vaccine, as well as the associated reporting of adverse events of special interest (AESI) post-administration of vaccines [30]. At the same time, the vaccination of related COVID-19 mRNA vaccines may cause the sequelae of myocarditis [8, 31-33], which brings vaccine hesitation and concerns about the safety of vaccines. From the countries/regions in Table **1** and Fig. (**2A**), the USA (126 articles) is the country with the highest productivity, far surpassing the second place England (46 articles). There is close collaboration between countries, especially China, with 10 times with England. In addition, according to Table **1** and Figs. (**2B** and **C**), the top 10 institutions are mainly from the USA, Israel, and the UK. The top 10 authors are mainly from the USA and Hong Kong. The reasons for the ranking of output and cooperation may be related to the population base, the degree of government attention, availability of data relative to the staged implementation of the vaccine (*e.g*., Israel/USA were first to roll out the vaccine at the population level), *etc*. [30]. Furthermore, through Fig. (**5**), we can know the authors with the same research direction, such as these five professors, Arthur Shiyovich, Guy Witberg, Ashraf Hamdan, Yaron Aviv, and Ran Kornowski, which provides references for cooperation and communication between authors. The cooperation between authors and institutions is concentrated. In addition, related studies were mostly published in Vaccines (27 articles), and other journals with high publication volumes were diverse.

### 4.2 Analysis of Research Hotspots and Frontier

Myocarditis is one of the adverse events related to the COVID-19 vaccines, and it refers to the inflammation of cardiac [34]. Billions of vaccines applied in more than 180 countries have proved their safety, and the incidence of adverse events is low [35, 36]. Generally speaking, the adverse events have the following characteristics: firstly, these complications greatly depend on age and sex [37-42]. Specifically, myocarditis clusters among 29.2-year-old middle men [41, 43]. As for different types of vaccines and their risks of myocarditis, several main types of vaccines include mRNA vaccines, adenovirus vector vaccines, and inactivated vaccines.

1. mRNA vaccines: mRNA vaccines have been heavily researched on account of their considerable application prospect and superiority, including short development cycle, easy industrialization, simple production process, flexibility to respond to new variants, the capacity to induce better immune response [30], and high effectiveness [44]. The majority of COVID-19 mRNA vaccines currently in clinical trials or already applicable are non-replicating mRNA vaccines, which have a significant advantage of the simple structure and shorter length substrate, whilst the optimized or modified one can greatly enhance the biological activity, including BNT162b2 cooperated by BioNTech and Pfizer [8, 30]. Additionally, according to Fig. (**7A**), in the year 2022, BNT162b2 may have constituted a frontier. BNT162b2 has substantial protection among people, including children [45] and older patients [46] during the Omicron period. It was administered intramuscularly as a two-dose scheme of 30 μg of immunogen per dose, 21 days apart. It has a prospective protection effect: 95% efficacy of preventing COVID-19 infection in individuals aged 16, and 92.6% even before the second dose. The second mRNA vaccine received EUA by the US government : mRNA-1273, developed by Moderna, encodes the entire length prefusion spike protein, is generally well tolerated and safe to use in adolescents and adults, has an efficacy of 94.1% [30]. However, the second dose of mRNA vaccine may be the trigger for myocarditis [47-49]: To be specific, myocarditis usually happens within a week of the BNT162b2 vaccine (Pfizer/BioNTech) or mRNA-1273 vaccine (Moderna) injection [9, 36, 50-53]. Additionally, an increased risk of myocarditis or pericarditis after the mRNA vaccine injection was found [50]. The safety of these two mRNA vaccines was compared: Wong *et al*. didn’t find a statistically significant difference in myocarditis or pericarditis risk between recipients of mRNA-1273 and BNT162b2 [35]. Abraham *et al*. reported a 1.6 to 5.0 times increased risk of myocarditis or pericarditis with mRNA-1273 vaccination compared with BNT162b2 vaccination [54]. Therefore, further studies are needed to explore their safety.
2. Adenovirus vector vaccines: ChAdOx1 nCoV-19 vaccine was safe and well tolerated with a lower reactogenicity profile [48]. Its side effects are basically mild to moderate and mainly include fever, fatigue, and headache. However, an increased risk of myocarditis is reported at 1–7 days following the first dose [55], whilst a decreased risk of pericarditis [50]. The probability of adverse events was comparable to that of the mRNA vaccine. MRNA had a higher incidence of acute cardiac injury, myocarditis, pericarditis, and arrhythmias than ChAdOx1 vaccinees. The incidence of mRNA was lower in Guillain-Barré syndrome, vasovagal syncope, radiculopathy, and aseptic arthritis [56]. It has also been shown that the ChAdOx1 vaccine is associated with an increased risk of thrombocytopenia and venous thromboembolism [57]. AD26.COV2.S is another widely used adenovirus-vectored vaccine that was authorized by the FDA on 2/27/2021 for use in persons 18 years of age and older [58]. In a side-by-side comparison of the three COVID-19 vaccines, BNT162b2 mRNA, mRNA-1273, and Ad26.COV2.S, reporting myocarditis or pericarditis after over a million vaccinated populations for each vaccine, Ad26.COV2.S had the latest onset of the time and the lowest reported rate [59]. The study also concluded that the reported rate of myocarditis and pericarditis after COVID-19 vaccination was less than 0.1%, and the reported rate of myocarditis and pericarditis secondary to vaccination remained lower than that of SARS-CoV-2 infection [59].
3. Inactivated vaccines: Compared with other COVID-19 vaccine candidates, killed vaccines’ including CoronaVac (by the Chinese company Sinovac), have the adjusted vaccine effectiveness of full immunization was 65.9% in a cohort study [60], country-level experience by Chile indicated a combination of poor effectiveness against mild or asymptomatic infection and waning effectiveness [61], so new vaccination options are substitutions. Compared with other sorts of vaccinations, inactivated vaccinations have the mildest adverse reactions, just with the most common symptom being injection-site pain [62]; myocarditis/pericarditis is hardly reported. A study in Malaysia demonstrated no association between the CoronaVac vaccine and all events except arrhythmia by identifying individuals vaccinated with COVID-19 and diagnosed with thrombocytopenia, venous thromboembolism, myocardial infarction, myocarditis/pericarditis, arrhythmia, stroke, Bell's Palsy, and convulsion/seizure in a self-controlled case series [57]. Furthermore, in another self-controlled case series and case-control study in Hong Kong, no significantly increased risk of carditis was observed after CoronaVac in all preliminary analyses [63]. In a record of Adverse events following immunization after 104.63 million vaccinations in Thailand, as compared with ChAdOx1-nCoV, BBIBP-CorV, BNT162b2, and mRNA-1273, CoronaVac also has the lowest reported rate of myocarditis/pericarditis [64]. At the immune level, CoronaVac and BNT162b2 have different durability and cross-variability protection, but CoronaVac recipients seem to have lower antibody titers than BNT162b2 [65].

All in all, vaccination-complicated myocarditis is considerably slight with a prospective prognosis: critical condition of myocarditis, including acute myocarditis, associated with a 12% rate of either in-hospital mortality or need for heart transplant is more likely to happen in unvaccinated, COVID-19 infectious people [50, 66, 67]. Chest pain, fever, myalgia/body aches, and abnormal electrocardiogram were frequently reported symptoms [43, 67].

Additionally, the diseases associated with the injection of COVID-19 vaccines and myocarditis are hot and at the forefront of this topic. This paper focuses on hypertension, heart failure, arrhythmias, and ischemic stroke. For hypertension, binding of the SARS-CoV-2 virus sarcomeric protein (S) to the cell surface angiotensin-converting enzyme 2 (ACE2) receptor mediates the entry of the virus into the cell, which results in a reduction in the amount of ACE2 and loss of ACE2 activity [68]. Vaccination produces free-floating S proteins with similar receptor-binding capacity to the natural SARS-CoV-2 S protein [69]. Whereas ACE2 is a critical enzyme in the conversion of angiotensin II (AngII) to angiotensin 1-7 (Ang1-7), down-regulation of ACE2 and an imbalance between AngII and Ang1-7 may directly contribute to the excessive elevation of blood pressure during the acute phase of SARS-CoV-2 infection and after COVID-19 vaccination [70].

Meanwhile, the accumulation of Ang II may enhance the pro-inflammatory cascade response induced by molecular mimicry between SARS-CoV-2 spicule proteins and self-antigens, leading to myocardial injury [71]. Post-vaccination COVID-19 vaccination may lead to both hypertension and myocarditis. However, the study suggests elevated blood pressure may be rare and transient [72]. Moreover, heart failure is a severe cardiac disease, and myocarditis is a risk factor for it. A study with a total study population of 101,786 patients showed that mRNA vaccination was not associated with an increased risk of heart failure, myocarditis, venous thromboembolism, or worsened all-cause mortality [73]. Moreover, advanced heart failure therapies, such as mechanical circulatory support devices, can be life-saving for rare cases where COVID-19 myocarditis progresses to severe disease [74]. Of the cardiac adverse events reported after COVID-19 vaccination, myocarditis is the most common. It has been implicated as a potential cause of bradyarrhythmias, and possible mechanisms by which COVID-19 vaccine leads to arrhythmias may include direct viral invasion *via* molecular mimicry/spiking (S) protein production, escalation of the inflammatory response, hypoxia, cardiomyocyte death, and ultimately scarring/fibrosis [75]. Furthermore, Ischemic stroke is one of the adverse events of the COVID-19 vaccine, along with myocarditis, with 10.9 ischemic strokes per 10,000 people in the BNT162b2 group [76]. A study based on the database of the National Health Insurance Service of Korea showed that ischemic stroke was significantly lower in fully vaccinated patients compared to unvaccinated patients [77].

Indeed, vaccinations may even have a protective effect among adverse events: SARS-CoV-2 vaccinations bring a lower risk of myocarditis in contrast with the infected condition. It can be seen that the epidemiology of myocarditis in relation to vaccination is a hotspot and frontier of research. However, the fact that epidemiology is at the forefront of this research has also led to the inappropriate use of some scientific data. Therefore, various factors may affect scientific data when it is disseminated to the public, including exaggerated reports and misunderstandings by the media. People show more significant anxiety about more serious words, such as death, severe, *etc*., even though their incidence in the overall vaccinated population may be extremely low. However, due to its potential severity and higher click-through rate, this information may be exaggerated on social media, leading to unnecessary panic and misinterpretation among the public. At the same time, some studies have shown that Italian Twitter moves around neutrality in the first half of 2021 and then steadily shifts to the negative zone [78]. Therefore, people and the media should remain cautious and objective when interpreting and disseminating scientific information.

### 4.3 Strength and Limitation

This study presents a pioneering, objective, accurate and comprehensive systematic analysis of COVID-19 vaccine and myocarditis publications and their trends, offering valuable insights for clinicians and scholars in the field. Through literature metrology and visual analysis, researchers can gain a clear understanding of the research hotspots, evolution, and development trends of the COVID-19 vaccine and myocarditis. Inevitably, there are some limitations to the study. Web of Science encompasses a wide range of high-quality journals, but there are still a few excellent non-English or non-indexed journals that may not have been included. Therefore, the identified articles may not fully represent all the studies on the COVID-19 vaccine and myocarditis. Further research endeavors should aim to address this limitation by conducting more detailed studies.

## CONCLUSION

In conclusion, from the annual publication volume of related literature based on the Web of Science SCI-E database, myocarditis related to the COVID-19 vaccine has attracted more and more attention to heart disease. China, the USA, and Europe have made the greatest contribution to this field, and the cooperation between them is closer, and the publications are more concentrated. The research focus and frontier are mainly on the relationship between myocarditis and different types of COVID-19 vaccines, the development of more types of vaccines in reducing the incidence and sequelae of COVID-19, but not with an increased incidence of myocarditis, and relief of vaccine hesitancy. These findings can help clinicians and researchers understand myocarditis and the COVID-19 vaccine research hotspots and provide references for future research directions.

## Figures and Tables

**Fig. (1):**
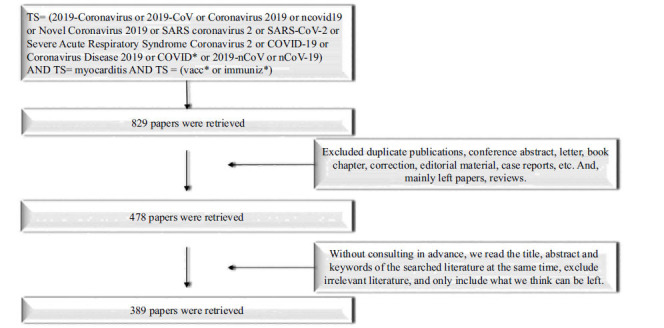
Flow chart of literature screening.

**Fig. (2):**
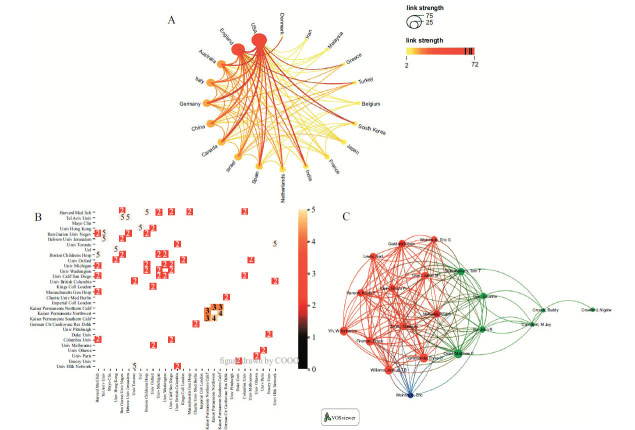
Countries/regions, institution, and author analysis. (**A**) Countries/regions cooperative network. (**B**) Institutions cooperative network. (**C**) Authors cluster map.

**Fig. (4):**
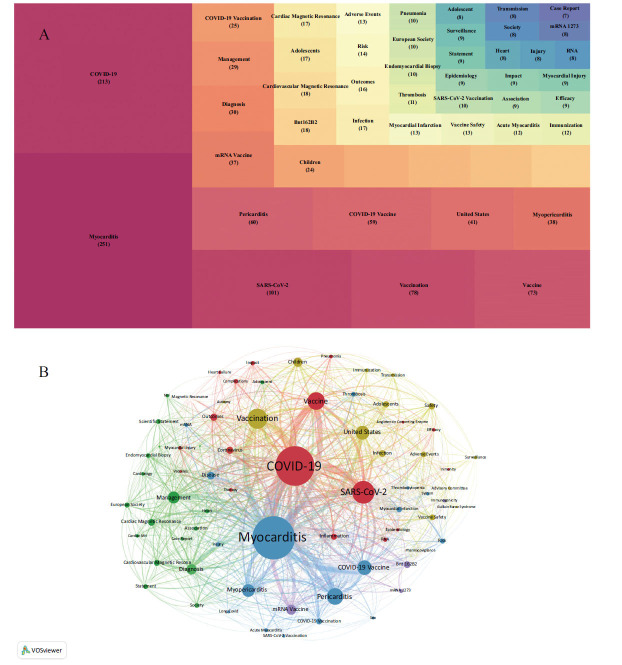
Keywords frequency and co-occurrence analysis (**A**) Tree map of top 50 keywords. (**B**) Keywords clustering analysis.

**Fig. (5):**
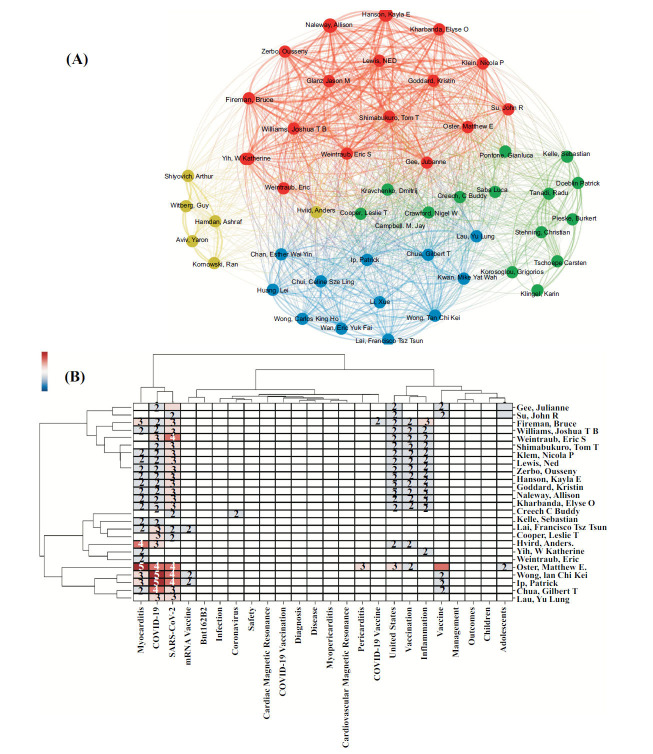
Keywords and authors analysis (**A**) Keywords and authors coupling matrix. (**B**) keywords and author two-modular matrix.

**Fig. (6):**
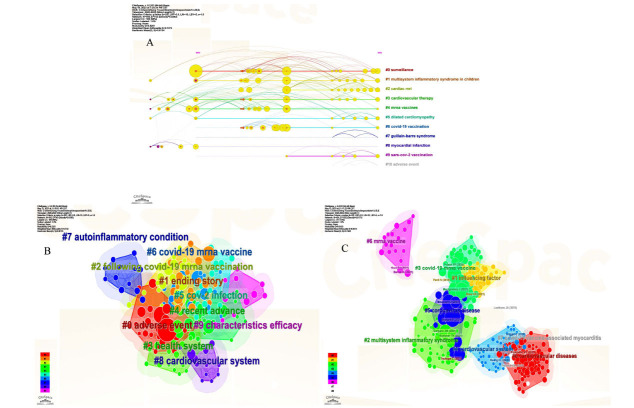
Cluster analysis (**A**) Timeline view of keywords (**B**) Cluster diagram of references using keywords in reference (**C**) Cluster diagram of references using title words in reference.

**Fig. (7):**
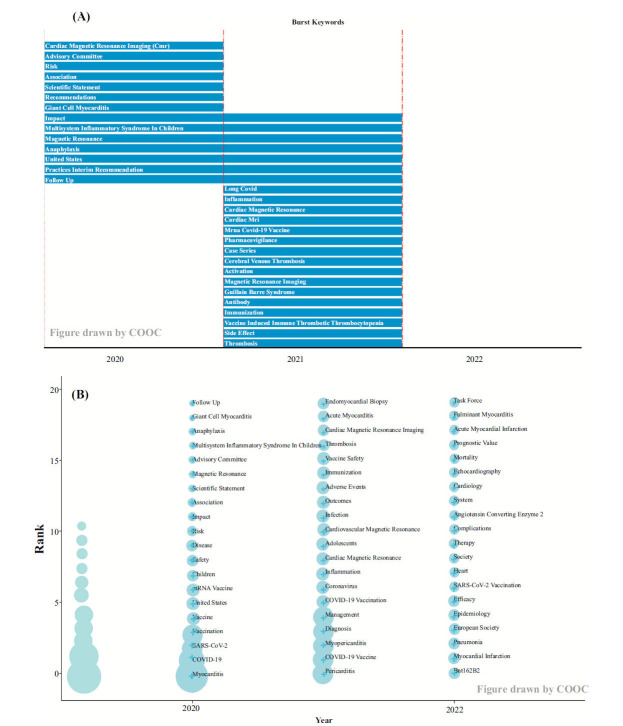
Keywords and time analysis. (**A**) Burst keywords map. (**B**) Time zone diagram of theme evolution path.

**Table 1:** Top 10 countries/regions, institutions, authors and journals.

**Rank**	**Country /Region**	**Count**	**Institution**	**Count**	**Author**	**Count**	**Journal**	**Count**	**2021 Impact Factor /JCR partition**
1	USA	126	Harvard Med Sch (USA)	13	Oster, Matthew E	8	Vaccines	27	4.961/Q2
2	England	41	Tel Aviv Univ (Israel)	13	Wong, Ian Chi Kei	6	Frontiers in Cardiovascular Medicine	23	5.846/Q2
3	Germany	35	Mayo Clin (USA)	9	Fireman, Bruce	5	Vaccine	12	4.169/Q3
4	Italy	34	Univ Toronto (Canada)	8	Ip, Patrick	5	Journal of Clinical Medicine	10	4.964/Q2
5	Japan	33	Ben Gurion Univ Negev (Israel)	8	Cooper, Leslie T	4	Journal of Korean Medical Science	8	5.354/Q2
6	China	31	UCL (UK)	8	Chua, Gilbert T	4	Frontiers in Immunology	8	8.786/Q1
7	Canada	25	Hebrew Univ Jerusalem (Israel)	8	Williams, Joshua T B	4	Clinical Infectious Diseases	7	20.999/Q1
8	South Korea	22	Univ Hong Kong (China)	8	Lai, Francisco Tsz Tsun	4	New England Journal of Medicine	5	176.079/Q1
9	Israel	22	Boston Childrens Hosp (USA)	7	Gee, Julianne	4	JAMA Cardiology	5	30.154/Q1
10	Australia	19	Univ Oxford (UK)	7	Klein, Nicola P	4	BMJ-British Medical Journal	5	93.333/Q1

**Table 2:** Ranking of the top 10 highest cited references.

**Rank**	**Year**	**Title**	**Journal**	**First Author**	**Citations**
1	2020	Cardiovascular Considerations for Patients, Health Care Workers, and Health Systems During the COVID-19 Pandemic	Journal of the American College of Cardiology	Driggin, Elissa	1153
2	2020	Potential Effects of Coronaviruses on the Cardiovascular System A Review	JAMA Cardiology	Madjid, Mohammad	1039
3	2020	The Science Underlying COVID-19 Implications for The Cardiovascular System	Circulation	Liu, Peter P	481
4	2021	Safety of the BNT162b2 mRNA COVID-19 Vaccine in A Nationwide Setting	New England Journal of Medicine	Barda, Noam	456
5	2022	Myocarditis with COVID-19 mRNA Vaccines	Circulation	Bozkurt, Biykem	386
6	2020	Myocarditis After COVID-19 Vaccination in a Large Health Care Organization	New England Journal of Medicine	Witberg, Guy	304
7	2021	Myocarditis Following Immunization with mRNA COVID-19 Vaccines in Members of the US Military	Jama Cardiology	Montgomery, Jay	298
8	2021	Myocarditis After BNT162b2 mRNA Vaccine Against COVID-19 in Israel	New England Journal of Medicine	Mevorach, D	288
9	2022	Comparing COVID-19 Vaccines for Their Characteristics, Efficacy and Effectiveness Against Sars-Cov-2 and Variants of Concern: A Narrative Review	Clinical Microbiology and Infection	Fiolet, Thibault	239
10	2021	Symptomatic Acute Myocarditis in 7 Adolescents After Pfizer-Biontech COVID-19 Vaccination	Pediatrics	Marshall, Mayme	220

**Table 3:** Ranking of the top 10 highest 180 days usage.

**Rank**	**Year**	**Title**	**Journal**	**First Author**	**Usage Count**
1	2022	2022 ACC Expert Consensus Decision Pathway on Cardiovascular Sequelae of COVID-19 in Adults: Myocarditis and Other Myocardial Involvement, Post-Acute Sequelae of SARS-CoV-2 Infection, and Return to Play a Report of the American College of Cardiology Solution Set Oversight Committee	Journal of the American College of Cardiology	Gluckman, Ty J	37
2	2022	Comparing COVID-19 Vaccines for Their Characteristics, Efficacy and Effectiveness Against SARS-CoV-2 and Variants of Concern: A Narrative Review	Clinical Microbiology and Infection	Fiolet, Thibault	37
3	2021	Myocarditis with COVID-19 mRNA Vaccines	Circulation	Bozkurt, Biykem	30
4	2022	Myopericarditis Following COVID-19 Vaccination and Non-COVID-19 Vaccination: A Systematic Review and Meta-Analysis	Lancet Respiratory Medicine	Ling, Ryan Ruiyang	23
5	2022	COVID-19 Vaccine Development: Milestones, Lessons and Prospects	Signal Transduction and Targeted Therapy	Li, Maochen	20
6	2020	Cardiovascular Considerations for Patients, Health Care Workers, and Health Systems During the COVID-19 Pandemic	Journal of the American College of Cardiology	Driggin, Elissa	18
7	2022	Myocarditis Cases Reported After mRNA-Based Covid-19 Vaccination in the US from December 2020 to August 2021	JAMA-Journal of the American Medical Association	Oster, Matthew E	15
8	2020	Potential Effects of Coronaviruses on the Cardiovascular System A Review	JAMA Cardiology	Madjid, Mohammad	15
9	2022	Risks of Myocarditis, Pericarditis, and Cardiac Arrhythmias Associated with COVID-19 Vaccination or SARS-CoV-2 Infection	Nature Medicine	Patone, Martina	14
10	2021	Safety of the Bnt162B2 mRNA COVID-19 Vaccine in A Nationwide Setting	New England Journal of Medicine	Barda, Noam	12

## Data Availability

The datasets generated during the current study are available in the Web of Science (http://www.webofknowledge.com).

## LIST OF ABBREVIATIONS

ACE2: Angiotensin-converting enzyme 2
AESI: Adverse event of special interest
COOC13.2: Co-occurrence13.2
COVID-19: Coronavirus disease 2019
JACC: Journal of the American college of cardiology
SCI-E: SCI-expanded
